# HPV Prevalence among Women from Appalachia: Results from the CARE Project

**DOI:** 10.1371/journal.pone.0074276

**Published:** 2013-08-30

**Authors:** Paul L. Reiter, Mira L. Katz, Mack T. Ruffin, Erinn M. Hade, Cecilia R. DeGraffenreid, Divya A. Patel, Electra D. Paskett, Elizabeth R. Unger

**Affiliations:** 1 Division of Cancer Prevention and Control, College of Medicine, The Ohio State University, Columbus, Ohio, United States of America; 2 Comprehensive Cancer Center, The Ohio State University, Columbus, Ohio, United States of America; 3 College of Public Health, The Ohio State University, Columbus, Ohio, United States of America; 4 Department of Family Medicine, University of Michigan, Ann Arbor, Michigan, United States of America; 5 Center for Biostatistics, The Ohio State University, Columbus, Ohio, United States of America; 6 Department of Obstetrics and Gynecology, University of Michigan, Ann Arbor, Michigan, United States of America; 7 Division of High-Consequence Pathogens and Pathology, National Center for Emerging and Zoonotic Infectious Diseases, Centers for Disease Control and Prevention, Atlanta, Georgia, United States of America; Vanderbilt University, United States of America

## Abstract

**Background:**

Cervical cancer incidence and mortality rates are high among women from Appalachia, yet data do not exist on human papillomavirus (HPV) prevalence among these women. We examined the prevalence of genital HPV among Appalachian women and identified correlates of HPV detection.

**Methods:**

We report data from a case-control study conducted between January 2006 and December 2008 as part of the Community Awareness, Resources, and Education (CARE) Project. We examined HPV prevalence among 1116 women (278 women with abnormal Pap tests at study entry [cases], 838 women with normal Pap tests [controls]) from Appalachian Ohio. Analyses used multivariable logistic regression to identify correlates of HPV detection.

**Results:**

The prevalence of HPV was 43.1% for any HPV type, 33.5% for high-risk HPV types, 23.4% for low-risk HPV types, and 12.5% for vaccine-preventable HPV types. Detection of any HPV type was more common among women who were ages 18–26 (OR = 2.09, 95% CI: 1.26–3.50), current smokers (OR = 1.86, 95% CI: 1.26–2.73), had at least five male sexual partners during their lifetime (OR = 2.28, 95% CI: 1.56–3.33), or had multiple male sexual partners during the last year (OR = 1.98, 95% CI: 1.25–3.14). Similar correlates were identified for detection of a high-risk HPV type.

**Conclusions:**

HPV was prevalent among Appalachian women, with many women having a high-risk HPV type detected. Results may help explain the high cervical cancer rates observed among Appalachian women and can help inform future cervical cancer prevention efforts in this geographic region.

## Introduction

Human papillomavirus (HPV) is the most common sexually transmitted infection (STI) in the United States (U.S.) [1], with infections having the potential to cause various adverse health outcomes. Among females, high-risk (i.e., oncogenic) HPV types, mainly types 16 and 18, cause over 90% of cervical and anal cancers and lower percentages of vaginal, vulvar, and oral cancers [2]. Low-risk (i.e., nononcogenic) HPV types, mainly types 6 and 11, cause almost all anogenital warts [3]. National estimates suggest that about 40% of females ages 14–59 are infected with at least one HPV type and about 30% are infected with a high-risk HPV type [4]. HPV prevalence differs across racial and ethnic groups, with infection more common among African American and Hispanic women [4]. The higher prevalence is likely a key factor contributing to the higher cervical cancer incidence rates observed among these racial and ethnic groups [5]. Thus, it becomes important to determine HPV prevalence among populations at high-risk for cervical cancer to better understand existing cervical cancer disparities.

A high-risk population that has not yet been examined in terms of HPV prevalence is women living in the Appalachian region of the U.S. Appalachia consists of the entire state of West Virginia and portions of 12 other states that extend from New York to Mississippi [6]. The region has a total population of about 25 million people (about 8% of the U.S. population) and is characterized by less racial diversity and lower socioeconomic status compared to the rest of the country [7]. Areas within Appalachia have among the highest cervical cancer incidence and mortality rates in the U.S. [8]. Research comparing Appalachian women to non-Appalachian women from the same states has shown cervical cancer incidence and mortality rates are often higher among the Appalachian women [9], [10].

There may be several reasons for the current cervical cancer disparities among women from Appalachia. Risky sexual behavior that likely increases the chance of HPV infection is common among these women [11]. Data also indicate that smoking rates are higher among Appalachian residents compared to their non-Appalachian counterparts from the same states [12], which is important since smoking may prolong the duration of HPV infections [13]. Furthermore, fewer Appalachian women receive cervical cancer screening tests compared to non-Appalachian women [14], [15]. The current study provides the first data we are aware of on the prevalence of HPV among women from Appalachia, which can help our understanding of the cervical cancer disparities among these women. This information is also essential for developing effective educational programs and interventions to address and reduce these disparities.

## Materials and Methods

### Ethics Statement

The Institutional Review Boards of The Ohio State University, the University of Michigan, and the Centers for Disease Control and Prevention (CDC) approved this study.

### Study Design

This study was part of the Community Awareness, Resources, and Education (CARE) Project, one of eight Centers for Population Health and Health Disparities (P50) funded by the National Institutes of Health (NIH). We report on one component of the CARE Project, a case-control study that focused on the determinants of abnormal cervical cytology among women living in Appalachian Ohio. A secondary aim of this study was to examine HPV prevalence among these women, the focus of this report. Appalachian Ohio is a 32 county region (29 at the time of this study) located in the southern and eastern sections of Ohio. Cervical cancer incidence and mortality rates are 12% and 21% higher, respectively, in Appalachian Ohio counties compared to the non-Appalachian counties in Ohio [10]. We conducted the current study between January 2006 and December 2008. Data from the CARE Project are available upon request.

Women included in this study were from 17 participating health clinics located throughout the Appalachian Ohio region. Women were eligible to participate if they were: 1) age 18 or older; 2) a resident of an Appalachian Ohio county; 3) had an intact uterine cervix and corpus; 4) were not pregnant; 5) did not have a history of cervical cancer; and 6) provided written informed consent. Women scheduled for a routine Papanicolaou (Pap) test on a day that a study nurse was at one of 17 clinics were asked to participate in the study. Women completed a self-administered questionnaire following consent and prior to undergoing their Pap test. During the visit, the provider took an additional research cytology sample and obtained a cervical sample in specimen transport medium (STM; Qiagen Inc., Valencia, CA) for HPV testing.

Women with abnormal cervical cytology at study entry were considered cases for the case-control study that aimed to identify the determinants of abnormal cervical cytology. Women were classified as having abnormal cervical cytology according to the 2001 Bethesda System for Reporting Pap Smear Results [16]. Using this system, cases were classified as having atypical squamous cells of undetermined significance (ASC-US), atypical squamous cells cannot exclude HSIL (ASC-H), atypical glandular cells (AGC), low-grade squamous intraepithelial lesions (LSIL), high-grade squamous intraepithelial lesions (HSIL), adenocarcinoma in situ (AIS), or carcinoma. We randomly selected three women with normal cervical cytology at study entry from the same clinic within a three-month window around the date of the case’s abnormal cervical cytology to serve as controls. This part of the CARE Project had a participation rate of 70% [17], with a total of 1131 women included in the case-control study. We report data on 1116 women (278 cases, 838 controls), excluding 15 women with inadequate HPV results.

### HPV Detection

The STM sample was stored at –70°C until shipped to the Centers for Disease Control and Prevention (CDC). At CDC, the samples were thawed and 150 µL of the 1.0 mL sample were extracted with the MagNA Pure DNA kit III (Roche Applied Science, Indianapolis, IN), with 10 µL of extract used in the Research Use Only (RUO) Linear Array (LA) HPV Genotyping Test (Roche Diagnostics, Indianapolis, IN). The LA assay detects 37 HPV types (6, 11, 16, 18, 26, 31, 33, 35, 39, 40, 42, 45, 51, 52, 53, 54, 55, 56, 58, 59, 61, 62, 64, 66, 67, 68, 69, 70, 71, 72, 73, 81, 82, 83, 84, 89, and IS39) and includes ß-globin as an endogenous positive control. Samples positive for the XR probe on the LA HPV strip that were also positive for HPV 33, 35, and 58 were tested with an HPV 52 quantitative PCR assay to confirm or exclude the presence of HPV 52 [18]. Samples negative for both ß-globin and HPV were considered inadequate for interpretation and omitted from analyses.

Based on HPV testing results, we examined detection of: 1) any HPV type; 2) a high-risk HPV type (16, 18, 26, 31, 33, 35, 39, 45, 51, 52, 53, 56, 58, 59, 64, 66, 67, 68, 69, 70, 73, 82, or IS39); 3) a low-risk HPV type (6, 11, 40, 42, 54, 55, 61, 62, 71, 72, 81, 83, 84, or 89); and 4) a vaccine-preventable HPV type (i.e., a type included in the quadrivalent HPV vaccine [19]: type 6, 11, 16, or 18). We used the same high- and low-risk classification scheme as a recent national study that analyzed data from the National Health and Nutrition Examination Survey (NHANES) [4] in order to facilitate comparisons between data from the CARE Project and the NHANES. For descriptive purposes, we also examined the detection of high-risk HPV types using a classification scheme from the International Agency for Research on Cancer (IARC)(types 16, 18, 31, 33, 35, 39, 45, 51, 52, 56, 58, 59, and 68; Groups 1 and 2A of the IARC scheme)[20]. Lastly, we examined the detection of each individual HPV type and detection of multiple HPV types (i.e., women testing positive for two or more HPV types). We do not, however, report detailed data on multiple-type HPV infections (e.g., HPV types present in the multiple-type infections). We feel such detailed data are beyond the scope of this manuscript, but the data will be considered for a future publication.

### Measures

We collected data on a wide range of health-related and demographic characteristics and examined them as potential correlates of HPV detection (Table 1). Data on potential correlates were obtained during the self-administered questionnaire that women completed prior to undergoing their cervical cancer screening test upon study entry.

**Table 1:** Characteristics of women from the CARE Project (n = 1116).

	n (%)
***Demographic Characteristics***	
Age (years)	
18–26	462 (41.5)
27–39	293 (26.3)
40 or older	358 (32.2)
Race/ethnicity	
Non-Hispanic white	1038 (93.9)
Other	67 (6.1)
Marital status	
Never married	362 (33.3)
Divorced/widowed/separated	199 (18.3)
Married/member of a couple	526 (48.4)
Education	
Less than a high school degree	131 (11.8)
High school degree	408 (36.7)
More than a high school degree	572 (51.5)
Annual household income	
$20,000 or less	477 (50.7)
$20,001–$50,000	297 (31.6)
More than $50,000	166 (17.7)
***Health Characteristics***	
Smoking status	
Never smoker	471 (42.6)
Former smoker	167 (15.1)
Current smoker	469 (42.4)
Ever been pregnant	
No	311 (28.3)
Yes	788 (71.7)
Pap test within the last 3 years	
No	161 (14.5)
Yes	949 (85.5)
Ever have prior abnormal Pap test	
No	620 (56.1)
Yes	486 (43.9)
Pap test result upon entry into CARE Project	
Normal (control)	838 (75.1)
Abnormal (case)	278 (24.9)
Ever told they had HPV infection (venereal warts, condylomas, or a papillomavirus infection)	
No	974 (88.5)
Yes	126 (11.5)
Age at first sexual intercourse	
15 or younger	350 (32.2)
16 or older	736 (67.8)
Number of male sexual partners during lifetime	
4 or fewer	458 (44.9)
5 or more	561 (55.1)
Number of male sexual partners during last year	
0–1	851 (79.5)
2 or more	220 (20.5)
Any male sexual partners with history of STI	
No/don’t know	952 (89.2)
Yes	115 (10.8)
Number of female sexual partners during lifetime	
0	1042 (95.2)
1 or more	53 (4.8)

*Note.* Totals may be less than stated sample size due to missing data. Percents may not sum to 100% due to rounding. CARE  =  Community Awareness Resources Education, HPV  =  human papillomavirus, STI  =  sexually transmitted infection.

We examined women’s smoking status (never smoker, former smoker, or current smoker) and whether they had ever been pregnant. We collected information on women’s cervical cancer screening histories and determined if they had a Pap test within the three years prior to study entry and whether they ever had a previous abnormal Pap test. Women indicated if they had ever been told by a doctor that they had an HPV infection (described to women as venereal warts, condylomas, or a papillomavirus infection). Women also indicated their age at first sexual intercourse, number of male sexual partners during their lifetime, number of male sexual partners within the last year, if any of their male partners had been diagnosed with an STI, and if they had any female sexual partners during their lifetime. We collected data on several demographic characteristics, including age, race/ethnicity, marital status, education, and annual household income. Age was categorized as 18–26 years old (within the recommended age range for HPV vaccination [19]), 27–39 years old, and 40 years or older.

### Statistical Analysis

We first calculated descriptive statistics in examining the prevalence of HPV among participants. We then used logistic regression to identify correlates of two outcomes: detection of any HPV type and detection of a high-risk HPV type (using the same classification scheme for high-risk HPV types as a recent study that analyzed data from the NHANES) [4]). For each outcome, we entered statistically significant univariable correlates (*p*<0.05) into a multivariable logistic regression model. Adjusted odds ratios (ORs) and 95% confidence intervals (CIs) were obtained from these multivariable logistic regression models. All logistic regression models included health clinic as a random effect since women were clustered within health clinics. Data were analyzed using SAS Version 9.2 (SAS Institute Inc., Cary, NC) and all statistical tests were two-tailed, using a critical value of α = 0.05.

## Results

### Participant Characteristics

Most women were less than 40 years old (67.8%), non-Hispanic white (93.9%), had at least a high school degree (88.2%), and reported a household income of $50,000 or less (82.3%)(Table 1). Less than half of women were married or a member of a couple (48.4%) and about a third indicated that they had never been married (33.3%). Over 40% of women (42.4%) were current smokers with an additional 15.1% being former smokers. A majority of women (85.5%) reported receiving a Pap test in the three years prior to study enrollment. Although 43.9% of women reported a history of at least one prior abnormal Pap test, only 11.5% indicated that a doctor had ever told them they had venereal warts, condylomas, or a papillomavirus infection. Most women reported they were age 16 or older at the time of their first sexual intercourse (67.8%) and just over half (55.1%) reported having at least five male sexual partners during their lifetime. Among women with an abnormal Pap test upon entry into the CARE Project (i.e., cases; n = 278), 143 (51.4%) had ASC-US, 7 (2.5%) had AGC, 112 (40.3%) had LSIL, 15 (5.4%) had HSIL, and 1 (0.4%) had squamous cell carcinoma.

### HPV Prevalence

The overall prevalence of any HPV type, as measured by HPV DNA detection in the LA assay, was 43.1% (Table 2). Using the same classification scheme as a recent study that analyzed data from the NHANES [4], a high-risk HPV type was detected in 33.5% of women while a low-risk HPV type as detected in 23.4% of women. Using the classification scheme from IARC [20], a high-risk HPV type was detected in 27.9% of women. A vaccine-preventable HPV type (6, 11, 16, or 18) was detected in 12.5% of women, including 21.0% of women ages 18–26. Among all women, HPV type-specific prevalence ranged from 0.0% to 8.2% (Table 3). The most prevalent HPV types included types 16 (8.2%), 53 (5.7%), 62 (4.8%), 51 (4.7%), 84 (4.6%), 89 (4.5%), 66 (4.3%), and 39 (4.1%). All other HPV types had a prevalence of less than 4.0%, including types 18 (2.7%), 6 (2.2%), and 11 (0.3%). Over 20% of women (22.4%) had two or more HPV types detected.

**Table 2:** HPV prevalence among women from the CARE Project and the NHANES.

	CARE – All women	CARE – Controls	CARE – Cases	NHANES^a^
	(n = 1116)	(n = 838)	(n = 278)	
Any HPV	43.1 (40.2–46.0)	32.1 (28.9–35.3)	76.3 (71.3–81.3)	39.2 (37.0–41.4)
High-risk HPV^b^	33.5 (30.7–36.3)	21.5 (18.7–24.3)	69.8 (64.4–75.2)	26.9 (24.7–29.1)
Low-risk HPV^c^	23.4 (20.9–25.9)	17.4 (14.8–20.0)	41.4 (35.6–47.2)	26.1 (24.0–28.2)

*Note.* Table reports percents and 95% confidence intervals in parentheses. HPV  =  human papillomavirus, CARE  =  Community Awareness Resources Education, NHANES  =  National Health and Nutrition Examination Survey.

a Estimates from the NHANES are for non-Hispanic white women [4], since 93.9% of women from the CARE Project were non-Hispanic white.

b Included HPV types 16, 18, 26, 31, 33, 35, 39, 45, 51, 52, 53, 56, 58, 59, 64, 66, 67, 68, 69, 70, 73, 82, and IS39 [4].

c Included HPV types 6, 11, 40, 42, 54, 55, 61, 62, 71, 72, 81, 83, 84, and 89 [4].

**Table 3:** Prevalence of HPV types among women from the CARE Project.

HPV type	Risk^a^	All women	Controls	Cases
		(n = 1116)	(n = 838)	(n = 278)
16	High	92 (8.2)	41 (4.9)	51 (18.3)
53	High	64 (5.7)	29 (3.5)	35 (12.6)
62	Low	53 (4.8)	21 (2.5)	32 (11.5)
51	High	52 (4.7)	18 (2.2)	34 (12.2)
84	Low	51 (4.6)	29 (3.5)	22 (7.9)
89	Low	50 (4.5)	22 (2.6)	28 (10.1)
66	High	48 (4.3)	19 (2.3)	29 (10.4)
39	High	46 (4.1)	18 (2.2)	28 (10.1)
54	Low	43 (3.9)	25 (3.0)	18 (6.5)
52	High	38 (3.4)	16 (1.9)	22 (7.9)
31	High	35 (3.1)	15 (1.8)	20 (7.2)
56	High	34 (3.1)	14 (1.7)	20 (7.2)
42	Low	31 (2.8)	17 (2.0)	14 (5.0)
59	High	31 (2.8)	13 (1.6)	18 (6.5)
18	High	30 (2.7)	13 (1.6)	17 (6.1)
61	Low	25 (2.2)	13 (1.6)	12 (4.3)
6	Low	24 (2.2)	10 (1.2)	14 (5.0)
45	High	22 (2.0)	9 (1.1)	13 (4.7)
67	High	22 (2.0)	13 (1.6)	9 (3.2)
83	Low	22 (2.0)	9 (1.1)	13 (4.7)
70	High	21 (1.9)	13 (1.6)	8 (2.9)
55	Low	19 (1.7)	10 (1.2)	9 (3.2)
35	High	18 (1.6)	11 (1.3)	7 (2.5)
58	High	18 (1.6)	9 (1.1)	9 (3.2)
68	High	18 (1.6)	8 (1.0)	10 (3.6)
73	High	18 (1.6)	7 (0.8)	11 (4.0)
40	Low	14 (1.3)	4 (0.5)	10 (3.6)
81	Low	14 (1.3)	9 (1.1)	5 (1.8)
82	High	11 (1.0)	6 (0.7)	5 (1.8)
33	High	7 (0.6)	0 (0.0)	7 (2.5)
72	Low	7 (0.6)	6 (0.7)	1 (0.4)
11	Low	3 (0.3)	2 (0.2)	1 (0.4)
26	High	1 (0.1)	0 (0.0)	1 (0.4)
71	Low	1 (0.1)	1 (0.1)	0 (0.0)
IS39	High	1 (0.1)	0 (0.0)	1 (0.4)
64	High	0 (0.0)	0 (0.0)	0 (0.0)
69	High	0 (0.0)	0 (0.0)	0 (0.0)

*Note.* Table reports n (%). HPV  =  human papillomavirus, CARE  =  Community Awareness Resources Education.

a High- and low-risk classification scheme is the same as a recent national study that analyzed data from the National Health and Nutrition Examination Survey (NHANES) [4].

### Correlates of HPV Detection

In multivariable analyses, detection of any HPV type was more common among women ages 18-26 compared to women ages 40 or older (OR = 2.09, 95% CI: 1.26–3.50)(Table 4). Current smokers were more likely to be HPV-positive compared to never smokers (OR = 1.86, 95% CI: 1.26–2.73). Detection of any HPV type was also more common among women with an abnormal Pap test upon entry into the CARE Project (OR = 5.59, 95% CI: 3.73–8.37), at least five male sexual partners during their lifetime (OR = 2.28, 95% CI: 1.56–3.33), or multiple male sexual partners during the last year (OR = 1.98, 95% CI: 1.25–3.14).

**Table 4:** Detection of any HPV type among women from the CARE Project (n = 1116).

	No. of women with any HPV type detected/total no. of women in category (%)	Univariable OR (95% CI)	Multivariable OR (95% CI)
***Demographic Characteristics***			
Age (years)			
18–26	268/462 (58.0)	3.02 (2.21–4.13)**	2.09 (1.26–3.50)*
27–39	110/293 (37.5)	1.40 (0.99–1.97)	1.00 (0.65–1.56)
40 or older	102/358 (28.5)	ref.	ref.
Race/ethnicity			
Non-Hispanic white	439/1038 (42.3)	ref.	--
Other	37/67 (55.2)	1.37 (0.82–2.31)	--
Marital status			
Never married	209/362 (57.7)	2.79 (2.08–3.74)**	1.26 (0.80–1.99)
Divorced/widowed/separated	96/199 (48.2)	2.02 (1.44–2.85)**	1.10 (0.69–1.76)
Married/member of a couple	159/526 (30.2)	ref.	ref.
Education			
Less than a high school degree	66/131 (50.4)	1.37 (0.92–2.02)	--
High school degree	189/408 (46.3)	1.24 (0.95–1.62)	--
More than a high school degree	223/572 (39.0)	ref.	--
Annual household income			
$20,000 or less	253/477 (53.0)	3.32 (2.14–5.17)**	1.66 (0.97–2.84)
$20,001–$50,000	110/297 (37.0)	1.88 (1.20–2.96)*	1.11 (0.66–1.87)
More than $50,000	37/166 (22.3)	ref.	ref.
***Health Characteristics***			
Smoking status			
Never smoker	155/471 (32.9)	ref.	ref.
Former smoker	63/167 (37.7)	1.24 (0.85–1.81)	0.98 (0.60–1.61)
Current smoker	259/469 (55.2)	2.19 (1.66–2.88)**	1.86 (1.26–2.73)*
Ever been pregnant			
No	164/311 (52.7)	ref.	ref.
Yes	310/788 (39.3)	0.62 (0.47–0.81)**	0.80 (0.50–1.27)
Pap test within the last 3 years			
No	65/161 (40.4)	0.85 (0.60–1.20)	--
Yes	413/949 (43.5)	ref.	--
Ever have prior abnormal Pap test			
No	230/620 (37.1)	ref.	ref.
Yes	249/486 (51.2)	1.74 (1.35–2.23)**	1.34 (0.95–1.90)
Pap test result upon entry into CARE Project			
Normal (control)	269/838 (32.1)	ref.	ref.
Abnormal (case)	212/278 (76.3)	6.81 (4.92–9.41)**	5.59 (3.73–8.37)**
Ever told they had HPV infection (venereal warts, condylomas, or a papillomavirus infection)			
No	401/974 (41.2)	ref.	ref.
Yes	73/126 (57.9)	1.99 (1.35–2.93)**	1.21 (0.73–2.00)
Age at first sexual intercourse			
15 or younger	174/350 (49.7)	1.35 (1.04–1.76)*	0.81 (0.56–1.16)
16 or older	299/736 (40.6)	ref.	ref.
Number of male sexual partners during lifetime			
4 or fewer	136/458 (29.7)	ref.	ref.
5 or more	314/561 (56.0)	2.87 (2.19–3.75)**	2.28 (1.56–3.33)**
Number of male sexual partners during last year			
0–1	307/851 (36.1)	ref.	ref.
2 or more	155/220 (70.5)	3.84 (2.74–5.38)**	1.98 (1.25–3.14)*
Any male sexual partners with history of STI			
No/don’t know	405/952 (42.5)	ref.	--
Yes	56/115 (48.7)	1.13 (0.76–1.68)	--
Number of female sexual partners during lifetime			
0	441/1042 (42.3)	ref.	ref.
1 or more	35/53 (66.0)	2.30 (1.25–4.21)*	0.79 (0.36–1.76)

*Note.* Totals may be less than stated sample size due to missing data. The multivariable model included data on 814 women due to missing data on correlates. The multivariable model did not include variables with dashes (--). HPV  =  human papillomavirus, CARE  =  Community Awareness Resources Education, OR  =  odds ratio, CI  =  confidence interval, ref.  =  referent group, STI  =  sexually transmitted infection.

* *p*<0.05, **p<0.001.

Multivariable correlates of high-risk HPV detection were highly similar to those for detection of any HPV type (Table 5). Detection of a high-risk HPV type was more common among women ages 18-26 compared to women ages 40 or older (OR = 3.25, 95% CI: 1.86–5.66) and among current smokers compared to never smokers (OR = 1.52, 95% CI: 1.01–2.30). Detection was also more common among women with a previous abnormal Pap test (OR = 1.51, 95% CI: 1.04–2.21), an abnormal Pap test upon entry into the CARE Project (OR = 6.26, 95% CI: 4.19–9.37), at least five male sexual partners during their lifetime (OR = 2.57, 95% CI: 1.69–3.92), or multiple male sexual partners during the last year (OR = 1.70, 95% CI: 1.07–2.69).

**Table 5:** Detection of high-risk HPV among women from the CARE Project (n = 1116).^a^

	No. of women with high-risk HPV type detected/total no. of women in category (%)	Univariable OR (95% CI)	Multivariable OR (95% CI)
***Demographic Characteristics***			
Age (years)			
18–26	227/462 (49.1)	4.56 (3.25–6.40)**	3.25 (1.86–5.66)**
27–39	85/293 (29.0)	1.97 (1.34–2.89)**	1.31 (0.80–2.16)
40 or older	61/358 (17.0)	ref.	ref.
Race/ethnicity			
Non-Hispanic white	341/1038 (32.9)	ref.	--
Other	28/67 (41.8)	1.27 (0.75–2.14)	--
Marital status			
Never married	172/362 (47.5)	2.80 (2.07–3.78)**	1.17 (0.73–1.88)
Divorced/widowed/separated	66/199 (33.2)	1.59 (1.10–2.31)*	0.97 (0.58–1.63)
Married/member of a couple	121/526 (23.0)	ref.	ref.
Education			
Less than a high school degree	50/131 (38.2)	1.25 (0.83–1.88)	--
High school degree	147/408 (36.0)	1.20 (0.91–1.59)	--
More than a high school degree	174/572 (30.4)	ref.	--
Annual household income			
$20,000 or less	190/477 (39.8)	3.17 (1.97–5.10)**	1.33 (0.73–2.43)
$20,001–$50,000	91/297 (30.6)	2.24 (1.37–3.66)*	1.21 (0.67–2.19)
More than $50,000	26/166 (15.7)	ref.	ref.
***Health Characteristics***			
Smoking status			
Never smoker	122/471 (25.9)	ref.	ref.
Former smoker	43/167 (25.8)	0.99 (0.66–1.51)	0.76 (0.44–1.34)
Current smoker	207/469 (44.1)	2.05 (1.54–2.73)**	1.52 (1.01–2.30)*
Ever been pregnant			
No	139/311 (44.7)	ref.	ref.
Yes	231/788 (29.3)	0.54 (0.41–0.71)**	0.75 (0.46–1.21)
Pap test within the last 3 years			
No	51/161 (31.7)	0.88 (0.60–1.27)	--
Yes	321/949 (33.8)	ref.	--
Ever have prior abnormal Pap test			
No	174/620 (28.1)	ref.	ref.
Yes	198/486 (40.7)	1.72 (1.33–2.23)**	1.51 (1.04–2.21)*
Pap test result upon entry into CARE Project			
Normal (control)	180/838 (21.5)	ref.	ref.
Abnormal (case)	194/278 (69.8)	8.35 (6.10–11.43)**	6.26 (4.19–9.37)**
Ever told they had HPV infection (venereal warts, condylomas, or a papillomavirus infection)			
No	314/974 (32.2)	ref.	ref.
Yes	57/126 (45.2)	1.73 (1.18–2.53)*	0.94 (0.55–1.61)
Age at first sexual intercourse			
15 or younger	141/350 (40.3)	1.42 (1.08–1.87)*	0.95 (0.64–1.40)
16 or older	228/736 (31.0)	ref.	ref.
Number of male sexual partners during lifetime			
4 or fewer	101/458 (22.1)	ref.	ref.
5 or more	253/561 (45.1)	2.77 (2.09–3.68)**	2.57 (1.69–3.92)**
Number of male sexual partners during last year			
0–1	230/851 (27.0)	ref.	ref.
2 or more	130/220 (59.1)	3.65 (2.65–5.03)**	1.70 (1.07–2.69)*
Any male sexual partners with history of STI			
No/don’t know	314/952 (33.0)	ref.	--
Yes	46/115 (40.0)	1.24 (0.82–1.86)	--
Number of female sexual partners during lifetime			
0	337/1042 (32.3)	ref.	ref.
1 or more	33/53 (62.3)	3.11 (1.73–5.60)**	1.23 (0.55–2.77)

*Note.* Totals may be less than stated sample size due to missing data. The multivariable model included data on 814 women due to missing data on correlates. The multivariable model did not include variables with dashes (--). HPV  =  human papillomavirus, CARE  =  Community Awareness Resources Education, OR  =  odds ratio, CI  =  confidence interval, ref.  =  referent group, STI  =  sexually transmitted infection.

a High-risk HPV types included types 16, 18, 26, 31, 33, 35, 39, 45, 51, 52, 53, 56, 58, 59, 64, 66, 67, 68, 69, 70, 73, 82, and IS39.

* *p*<0.05, **p<0.001.

## Discussion

Appalachia is a geographic area with high cervical cancer incidence and mortality rates [8]–[10], yet the prevalence of genital HPV among Appalachian women is not known. Determining this prevalence is necessary to better understand the existing cervical cancer disparities among women from Appalachia. We detected at least one HPV type in over 40% of women, and detection of high-risk HPV was more common than detection of low-risk HPV. When our data are compared to HPV prevalence estimates among non-Hispanic white women from the NHANES [4](since our study sample was predominantly non-Hispanic white), we believe our findings potentially shed new light on current cervical cancer disparities (Table 2). Appalachian women from our study were similar to non-Hispanic white women from the NHANES in terms of the prevalence of any HPV type (43.1% [95% CI: 40.2%–46.0%] vs. 39.2% [95% CI: 37.0%–41.4%] [4]) and low-risk HPV (23.4% [95% CI: 20.9%–25.9%] vs. 26.1% [95% CI: 24.0–28.2] [4]). However, the prevalence of high-risk HPV was higher among Appalachian women (33.5% [95% CI: 30.7%–36.3%] vs. 26.9% [95% CI: 24.7–29.1] [4]). This pattern of findings is important because high-risk HPV types have a causal role in almost all cervical cancers [21], and the higher prevalence of these HPV types among Appalachian women may be a key contributing factor to the higher cervical cancer incidence rates observed among these women.

The design of our study should be considered when interpreting these results. Our data are from a case-control study that was designed to identify determinants of abnormal cervical cytology, so one-fourth of our sample had an abnormal Pap test upon study entry. This is higher than the prevalence of abnormal cytology among women from studies that were not case-control studies (up to 14% [22]). The prevalence of HPV in our study may therefore differ from what would be obtained from a random sample of Appalachian women. Indeed, HPV detection was more common among the cases in our study compared to controls. We do not believe, however, that study design solely accounts for the pattern of findings observed in the current study. Although future research is needed to confirm the findings from this study, our data provide valuable initial insight into the prevalence of HPV among Appalachian women and suggest that high-risk HPV infections may be more prevalent among women from this region, which may help explain the existing cervical cancer disparities in Appalachia.

Detection of any HPV type and detection of a high-risk HPV type were more common among women ages 18–26 compared to older women in our study. This pattern is similar to what has been found in past studies, where HPV was more prevalent among younger women [4]. Importantly, a vaccine-preventable HPV type was detected in over 20% of women ages 18–26, which is higher than the prevalence of these HPV types among women of similar ages from the NHANES (8.8% [23]). These results suggest that Appalachian females could benefit substantially from HPV vaccine. HPV vaccine is available in most healthcare facilities in Appalachia [24], and adults in the Appalachian region are accepting of the vaccine for females [25], [26]. However, HPV vaccine uptake among adolescent females varies greatly within the Appalachian region and is especially lacking in regions of Appalachia with existing cervical cancer disparities [27], [28]. Since HPV vaccine uptake tends to be even lower among young adult females compared to adolescent females [29], [30], it is likely that most young adult women in Appalachia remain unvaccinated. Our data begin to demonstrate the potential impact that HPV vaccination can have among these young women, and future efforts are needed to ensure that HPV vaccine uptake is occurring among this age group.

Detection of any HPV type and detection of a high-risk HPV type was more common among women who were current smokers or reported a higher number of sexual partners (both lifetime and recent partners). These findings coincide with those from previous studies examining HPV prevalence among women in the U.S. [4], [31]. Risky sexual behavior, such as a higher number of sexual partners, increases the risk of exposure to HPV [32], and smoking may prolong the duration of HPV infections and decrease the chance of clearing infections [13]. These issues could be particularly relevant when examining HPV prevalence among Appalachian women, as data indicate that risky sexual behavior is common among these women [11] and smoking rates are higher among Appalachian residents compared to non-Appalachian residents [12]. Thus, risky sexual behavior and smoking are likely playing an important role in Appalachian women acquiring an HPV infection and the subsequent risk of developing an HPV-related disease. Knowledge about HPV and cervical cancer risk factors is lacking among Appalachian women [26], [33], so educational programs are needed to increase these women’s awareness of and knowledge about these issues.

Study strengths include a large sample size and inclusion of women from throughout the Appalachian Ohio region. Our study also has several limitations. We were not able to examine the persistence or clearance of HPV infection. The study included women presenting to health clinics, which may limit the generalizability of findings since these women may have greater access to Pap testing compared to others from Appalachian Ohio. This may be important since Pap testing within recommended guidelines tends to be less common among women from Appalachian Ohio compared to women from non-Appalachian Ohio [34]. It is also possible that some women in our study were being seen at the health clinics for reasons related to HPV infection. Similar to the NHANES [4], participants self-reported behavioral data (e.g., sexual behavior and smoking status), which may have resulted in some misclassification. HPV vaccination data were available on less than 20% of women in our study, which did not allow us to account for this variable in analyses. However, less than 6% of women for whom vaccination data were available had received any doses of HPV vaccine, suggesting the impact of vaccination on our results was likely minimal.

HPV was prevalent among Appalachian women and many had a high-risk HPV type detected. These data not only provide the first insight into the prevalence of HPV among Appalachian women but may also help explain the existing cervical cancer disparities within this geographic region. Results can help inform future cervical cancer prevention efforts in Appalachia, specifically those designed to increase HPV vaccine uptake or women’s awareness of and knowledge about HPV and cervical cancer.

### Disclaimer

The findings and conclusions in this report are those of the authors and do not necessarily represent the views of the funding agencies.
