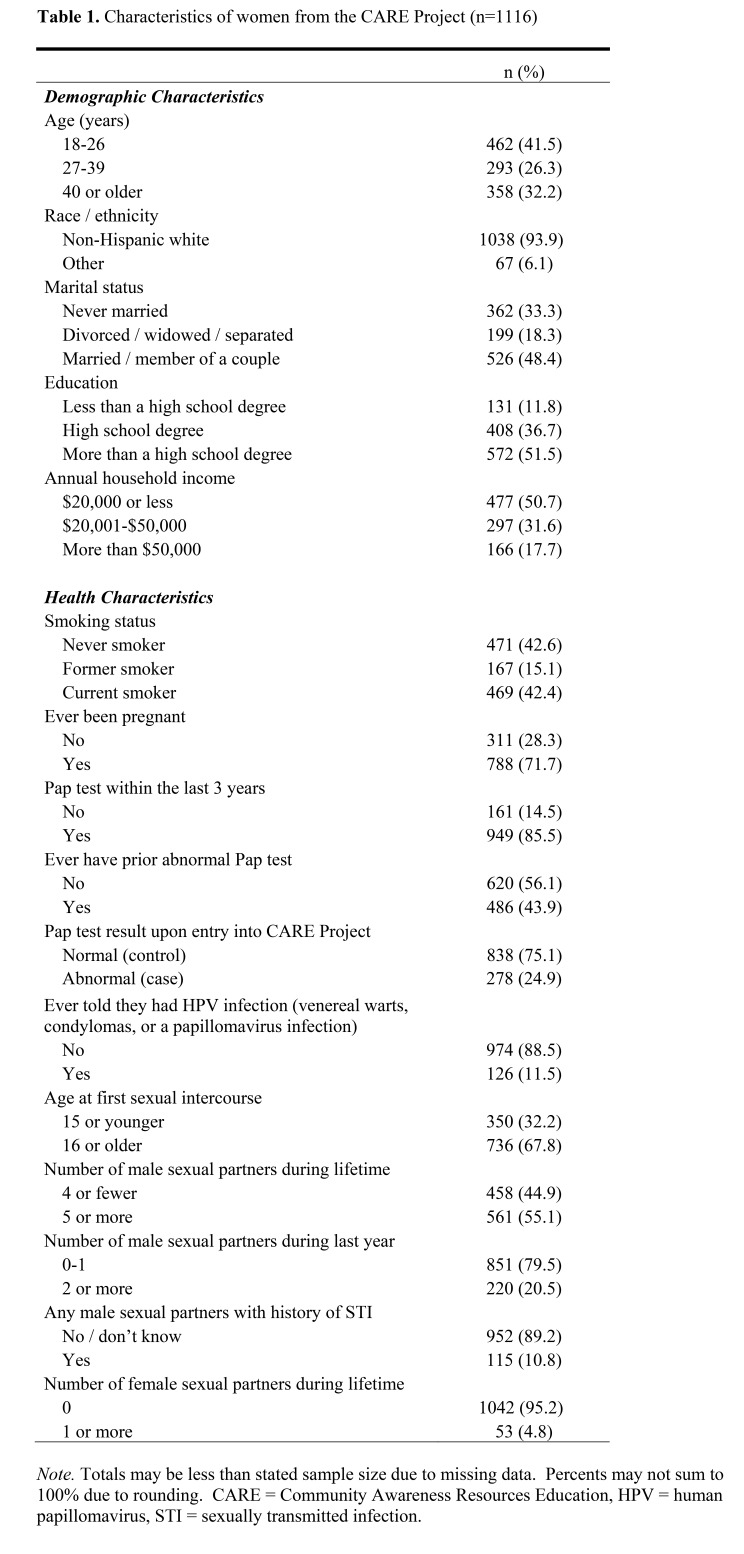# Correction: HPV Prevalence among Women from Appalachia: Results from the CARE Project

**DOI:** 10.1371/annotation/7a4cfd0a-cf77-430e-89a0-b2576268e2eb

**Published:** 2013-10-25

**Authors:** Paul L. Reiter, Mira L. Katz, Mack T. Ruffin, Erinn M. Hade, Cecilia R. DeGraffenreid, Divya A. Patel, Electra D. Paskett, Elizabeth R. Unger

Table 1 was incorrectly a duplicate of Table 3. The correct version of Table 1 is available here: 

**Figure:**